# Spontaneous Remission and Concomitant Progression in a Patient with DLBCL

**DOI:** 10.3390/diagnostics10110950

**Published:** 2020-11-14

**Authors:** Eun Ji Han, Jihyun Kim, Suk Young Park, Joo Hyun O

**Affiliations:** 1 Division of Nuclear Medicine, Department of Radiology, College of Medicine, The Catholic University of Korea, Seoul 06591, Korea; iwao@caholic.ac.kr (E.J.H.); march4900@hanmail.net (J.K.); 2 Division of Hematooncology, Department of Internal Medicine, College of Medicine, The Catholic University of Korea, Seoul 06591, Korea; sypark1011@hotmail.com

**Keywords:** diffuse large B-cell lymphoma, spontaneous remission, FDG, PET/CT

## Abstract

Diffuse large B-cell lymphoma (DLBCL) is the most common type of lymphoma. Although DLBCL can be cured in more than half of all patients, up to 50% of patients become refractory to initial treatment or relapse after complete remission. We present a case of complete spontaneous remission of some tumors and concomitant newly developed tumors observed in a patient with relapsed DLBCL. Spontaneous remission of lymphoma without treatment is a rare phenomenon and can occur at baseline as well as in relapsed DLBCL. However, most patients who initially experience spontaneous remission later develop relapse. Thus, careful follow-up is required, and fluorine-18-fluorodeoxyglucose (^18^F-FDG) positron emission tomography (PET)/computed tomography (CT) allows monitoring of multiple lesions.

Diffuse large B-cell lymphoma (DLBCL) is the most common non-Hodgkin’s lymphoma (NHL) accounting for approximately 30% of all cases worldwide [1]. The standard first-line treatment for de novo DLBCL is the combination of rituximab, cyclophosphamide, hydroxydaunorubicin, vincristine, prednisone (R-CHOP) chemotherapy [2]. Although DLBCL can be cured with first-line chemotherapy in more than half of all patients, up to 50% of patients do not respond to initial treatment or relapse after showing preliminary response [3]. Spontaneous remission of lymphoma without treatment is an uncommon phenomenon and is reported with varying range. In low grade lymphoma, reported incidence of spontaneous remission is as high as 10% to 23%, but the incidence is much lower in high grade lymphoma [4,5,6]. The mechanism of remission is not clearly understood, but tumor-associated molecular and patient-induced immunologic mechanisms (immunomodulatory change) or hormonal changes may have roles [7,8]. Previous DLBCL cases of spontaneous remission report broad range of time from diagnosis to remission from 15 days to 240 days. As seen in this case (Figure 1), spontaneous remission can occur in relapsed DLBCL as well. In the same patient, some lesions may show complete remission, while discordant disease progression can occur in other lesions. The literature observed that, for most patients who experience spontaneous remission, eventual relapse is inevitable, as it was in our case [7,9,10]. Thus, careful follow-up is required, and ^18^F-FDG PET/CT can aid in surveillance and tracking of multiple lesions.

## Figures and Tables

**Figure 1:**
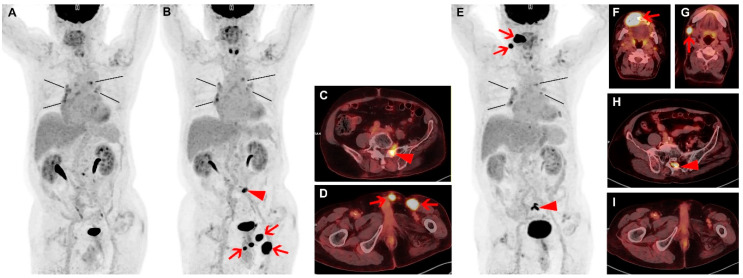
A 67-year-old male patient was diagnosed with diffuse large B-cell lymphoma (DLBCL) and had right orchiectomy followed by 8 cycles of rituximab, cyclophosphamide, hydroxydaunorubicin, vincristine, prednisone (R-CHOP) chemotherapy, and then radiotherapy, and complete response was achieved. Three years after the first line of therapy, the patient experienced relapse confirmed by left orchiectomy. Six cycles of dexamethasone, L-asparaginase, ifosfamide, carboplatin, etoposide (DL-ICE) chemotherapy, and radiotherapy followed, and the end-of-therapy fluorine-18-fluorodeoxyglucose (^18^F-FDG) positron emission tomography (PET)/ computed tomography (CT) findings indicated complete metabolic response again (**A**). FDG uptakes in the mediastinum and the peribronchial nodes showed similar patterns as in the previous ^18^F-FDG PET/CT studies without any size change and were assumed to be reactive nodal hyperplasia (**A**; black lines). Four months after the second line of therapy, the patient developed a palpable mass in left orchiectomy bed. Restaging ^18^F-FDG PET/CT demonstrated newly developed intense focal FDG uptakes in left orchiectomy bed (SUVmax 22.7), left inguinal area, and penis (**B**,**D**; arrows). In addition, localized FDG uptake was newly noted in sacrum and adjacent left L5/S1 intervertebral foramen (SUVmax 10.8; **B**,**C**; arrow heads). Clinically, these new lesions were considered to be second relapse of lymphoma. The patient refused further treatment and was lost to follow-up. Seven months later, the patient returned to the hospital complaining of discomfort in the oral cavity. Biopsy from right gingival lesion was performed. Immunohistochemistry confirmed tumor cells to be positive for CD79 and CD20 and negative for CD3, and the histology confirmed relapsed DLBCL. ^18^F-FDG PET/CT was performed for evaluation of overall disease status (**E**). Previously noted intense FDG uptakes in left orchiectomy bed, left inguinal area, penis, and sacrum had disappeared (**E**,**I**), without any form of treatment according to the patient and available medical records. Through multiple interviews, the patient and his family corroborated that the patient did not receive care at outside hospital. However, intense FDG uptakes were newly noted in right upper gingiva and adjacent maxilla (SUVmax 29.8; **E**,**F**; arrows), right cervical lymph node (**E**,**G**; arrows), and along the sacral canal at S2 level (SUVmax 12.3; **E**,**H**; arrow heads). FDG uptakes in the mediastinum and the peribronchial nodes showed little interval change (**A**,**B**,**E**; black lines).

## References

[1] Li S., Young K.H., Medeiros L.J. (2018). Diffuse large B-cell lymphoma. Pathology.

[2] Iacoboni G., Zucca E., Ghielmini M., Stathis A. (2018). Methodology of clinical trials evaluating the incorporation of new drugs in the first-line treatment of patients with diffuse large B-cell lymphoma (DLBCL): A critical review. Ann. Oncol..

[3] Crump M., Neelapu S.S., Farooq U., Van Den Neste E., Kuruvilla J., Westin J., Link B.K., Hay A., Cerhan J.R., Zhu L. (2017). Outcomes in refractory diffuse large B-cell lymphoma: results from the international SCHOLAR-1 study. Blood.

[4] Gattiker H.H., Wiltshaw E., Galton D.A. (1980). Spontaneous regression in non-Hodgkin’s lymphoma. Cancer.

[5] Kumar R., Bhargava P., Zhuang H., Yu J.Q., Schuster S.J., Alavi A. (2004). Spontaneous regression of follicular, mantle cell, and diffuse large B-cell non-Hodgkin’s lymphomas detected by FDG-PET imaging. Clin. Nucl. Med..

[6] Kumamoto M., Nakamine H., Hara T., Yokoya Y., Kawai J., Ito H., Nishioka S., Takenaka T., Wickert R.S., Mitchell D.W. (1994). Spontaneous complete regression of high grade non-Hodgkin’s lymphoma. Morphologic, immunohistochemical, and gene amplification analyses. Cancer.

[7] Pasvolsky O., Berger T., Bernstine H., Hayman L., Raanani P., Vidal L. (2019). Spontaneous Regression of Hodgkin Lymphoma: Case Report and Review of the Literature. Acta Haematol..

[8] Snijder J., Mihyawi N., Frolov A., Ewton A., Rivero G. (2019). Spontaneous remission in diffuse large cell lymphoma: a case report. J. Med. Case Rep..

[9] Sasaki J., Kurihara H., Nakano Y., Kotani K., Tame E., Sasaki A. (2016). Apparent spontaneous regression of malignant neoplasms after radiography: Report of four cases. Int. J. Surg. Case Rep..

[10] Ramos R., Fernandes J.S., Almeida M., Almeida R. (2018). A Rare Case of Spontaneous Remission and Relapse of a Primary Central Nervous System Lymphoma. Acta Med. Port..

